# Reducing Maternal Mortality and Improving Maternal Health: Bangladesh and MDG 5

**DOI:** 10.3329/jhpn.v26i3.1896

**Published:** 2008-09

**Authors:** Marge Koblinsky, Iqbal Anwar, Malay Kanti Mridha, Mahbub Elahi Chowdhury, Roslin Botlero

**Affiliations:** 1 ICDDR, B, GPO Box 128, Dhaka 1000, Bangladesh; 2 Women's Health Program, Department of Medicine, Monash University, Melbourne, Australia

**Keywords:** Maternal health, Maternal mortality, Obstetric care, Quality of care, Bangladesh

## Abstract

Bangladesh is on its way to achieving the MDG 5 target of reducing the maternal mortality ratio by three-quarters between 1990 and 2015, but the annual rate of decline needs to triple. Although the use of skilled birth attendants has improved over the past 15 years, it remains less than 20% as of 2007 and is especially low among poor, uneducated rural women. Increasing the numbers of skilled birth attendants, deploying them in teams in facilities, and improving access to them through messages on antenatal care to women, have the potential to increase such use. The use of caesarean sections is increasing although not among poor, uneducated rural women. Strengthening appropriate quality emergency obstetric care in rural areas remains the major challenge. Strengthening other supportive services, including family planning and delayed first birth, menstrual regulation, and education of women, are also important for achieving MDG 5.

## INTRODUCTION

Bangladesh, with the third largest number of poor people in the world after China and India, signed onto achieving the Millennium Development Goals (MDGs) by 2015. National policies and programme implementation over the past decade and a half follow the commitment, specifically for MDG 5 (improving maternal health). Beginning in 1994, the emergency obstetric care (EmOC) approach dominated with assistance from the United Nations Children's Fund (UNICEF), United Nations Population Fund (UNFPA), and the Averting Maternal Death and Disability programme in the renovation and upgradation of existing facilities and training of facility staff. With the development of the National Maternal Health Strategy in 2001, the approach broadened, building on the rights’ approach for safer motherhood and was incorporated into the ongoing Health and Population Sector Programme (HPSP) and subsequently into the Health, Nutrition and Population Sector Programme (HNPSP), the programmes that lay out the policies and programmes for government services. Delivery of interventions was made through the one-stop essential services package (ESP) at the primary healthcare level with health and family-planning cadres unified under one management structure. While the ESP had five components, maternal health had the highest priority, with a focus on EmOC to reduce the maternal mortality ratio and basic obstetric care for the promotion of good practices and for the early detection of complications and appropriate referral. Even when the Government moved away from the unified management structure and one-stop ESP in 2001, the focus remained the same.

To complement the facility approach to obstetric care, a skilled birth attendant strategy was initiated in 2001 with guidance from the World Health Organization (WHO) and UNFPA. The skilled birth attendants (SBAs) are to provide normal safe delivery in homes and referral to the EmOC sites, if needed. Drawing from the pool of 24,000 field-based female health and family-planning workers, the Government of Bangladesh (GoB) began training of community SBAs (CSBAs) for six months at the district level. By the end of 2007, about 3,000 had been trained; a further 1,000 are to be trained yearly to achieve complete coverage, theoretically in a decade.

**Key messages**

- Bangladesh is on its way to achieving the MDG 5 target of reducing the maternal mortality ratio by three-quarters between 1990 and 2015, but the annual rate of decline needs to triple.
- Use of skilled birth attendants has not improved in over a decade, especially among poor, uneducated rural women. Increasing the numbers of skilled birth attendants, deploying them in teams in facilities, and improving access to them through messages on antenatal care to women have the potential to increase such use.
- Use of caesarean sections is increasing, although not among poor, uneducated rural women. Strengthening appropriate quality emergency obstetric care in rural areas remains a major challenge.
- Strengthening other supportive services, including family planning and delayed first birth, menstrual regulation, and women's education, are important for reaching MDG 5.

The GoB's programmatic strategies to achieve MDG 5, EmOC at the facility level, and CSBAs providing safe delivery care at home are the subject of this paper. Achieving Goal 5 is measured through two globally-set indicators—reduction of the maternal mortality ratio by three quarters between 1990 and 2015 and an increased proportion of births attended by skilled health personnel. Progress made in attaining the two indicators through the strategies and projection to 2015 is reviewed.

## MDG 5: IMPROVING MATERNAL HEALTH: PROGRESS TOWARDS THE INDICATORS

### Indicator 1—Reduce the maternal mortality ratio

**Fig. 1:**
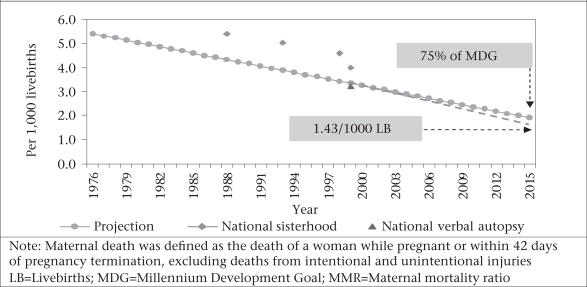
Projection of MMR decline in Bangladesh during 1976–2015

Bangladesh is considered to be on its way towards achieving the MDG 5 target of reducing the maternal mortality ratio (MMR) by three-quarters between 1990 and 2015. Since 1990, the MMR in Bangladesh has declined from 514 in 1986–1990 to 400 in 2003—22% in the 11 intervening years (1). Since these data comprise pregnancy-related deaths which include accidental/injury-related deaths and maternal deaths, these are higher than the MMR. Verbal autopsies of pregnancy-related deaths estimate ‘true’ maternal deaths at 322 (confidence interval [CI] 253-391) at the national level for the 1998–2000 period—20% lower than the pregnancy-related death estimate. Given these rates, approximately 11,000-12,000 maternal deaths occur each year in Bangladesh. While there has been progress, the rate of the decline of MMR must increase three-fold over the remaining 15 years to achieve the government-targeted level of 143 by 2015 (Fig. 1).

#### Causes and timing of maternal deaths

Based on the national maternal mortality survey, approximately 85% of maternal deaths in Bangladesh result from direct obstetric causes. These are primarily haemorrhage among women aged over 25 years and eclampsia among younger women aged 15-24 years (1). Data from the Matlab surveillance system confirm these findings with 82% of maternal deaths in Matlab during 1990–2001 directly caused by the major obstetric complications, haemorrhage being the primary culprit (antepartum haemorrhage [APH] and postpartum haemorrhage contributing 27% together), followed by eclampsia/pre-eclampsia with 23% (2). Similar to the findings of the national survey, younger women tend to die more from pregnancy-induced hypertension (PIH) compared to older women [during 1995–2001, 34%, 48%, and 18% died from pregnancy-induced hypertension (PIH) in the age-groups of ≥19, 20-29, and ≥30 years) respectively, and older women more frequently die from haemorrhage (168 women died from haemorrhage with 13%, 41%, and 46% at ≥19, 20-29, and ≥30 years of age respectively)].

**Fig. 2:**
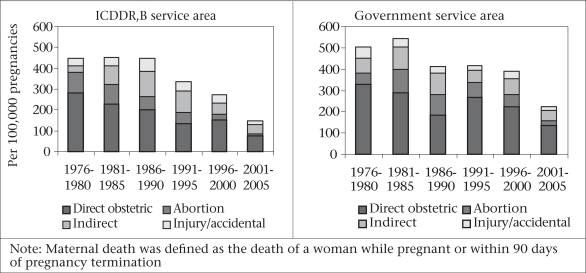
Trends in pregnancy-related deaths by types in ICDDR, B and government service areas in Matlab, 1976–2005

Deaths from induced abortion have declined in both Matlab intervention area* and adjacent government service area when the 1995–2005 level is compared with the pre-intervention levels of 1976–1980; however, the former is much more pronounced (88% decline in the Matlab intervention area vs 55% in the government area). Because of the successful family-planning programme at the national level and legal menstrual regulation available through government services, it is likely that the national levels of abortion-related maternal deaths have fallen over the years as well (Fig. 2).

The ICD-10 definition of maternal mortality excludes deaths due to accidents and injuries. However, 20% of deaths among pregnant unmarried women in Matlab were due to suicide compared to 5% for married women, and pregnant girls were nearly three times more likely to die from violent causes than non-pregnant girls (3,4). Obviously, pregnancy is a risk factor for their deaths.

Maternal mortality is extremely high during labour, on the delivery day, and within 48 hours after delivery. In Matlab, for example, 40% of maternal deaths from all causes occurred at these times during 1976–1985 prior to any intervention (5).

#### Place of death

With most deliveries in the home, it is not surprising that, during 1998–2001, most maternal deaths were also in the home: 45% in the husband's home, and 28% in the woman's natal home. However, 18% were in health facilities while nearly 5% were in transit (1). Since only 9% of all births were in a facility, this suggests that women are beginning to seek professional care when problems are recognized.

#### Fertility change and its contribution to reduction of maternal deaths

While MDG 5 is a measure of the overall risk of maternal mortality per 100,000 livebirths, the risk to individual groups of mothers differ, depending on age and parity. Thus, changing fertility can also reduce the numbers of maternal deaths by reducing the proportion of deliveries that are at higher risk. Bangladesh is considered a model country in reducing fertility—total fertility was 6.6 per woman aged 15-49 years in the mid-1970s and 3.0 in 2004 (6). With this reduction in fertility, there is a corresponding reduction in the number of pregnancies, which place the women at risk of maternal death. For example, in the ICDDR, B field area in Matlab which has had a major reduction in fertility during 1983–2001, about one-third of the reduction in maternal deaths was due to the reduction in fertility. Of this reduction, one-quarter was due to the changed pattern of childbearing (changing parity and age of the mother at time of delivery) and three-quarters were due simply to the lower level of fertility.

**Fig. 3:**
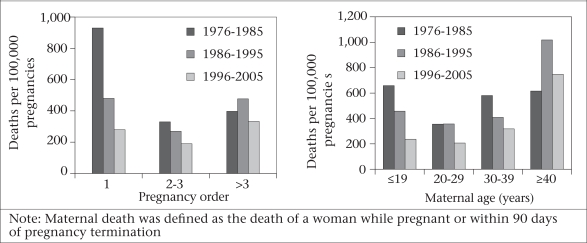
Trends in pregnancy-related deaths by pregnancy order, Matlab, 1976–2005

Paralleling the decline in fertility, the age at first birth in the Matlab intervention area shifted the higher risk from ≤19 years to 20-29 years old women. Death in both age-groups has decreased over time, but more so for the ≤19-year age-group. In 1976–1985, the mortality rate among women aged ≤19 years was 659 per 100,000 pregnancies; this rate declined to 237 per 100,000 pregnancies in 1996–2005. For women within the 20-29 years age-group, the corresponding reduction was from 355 to 207 per 100,000 pregnancies. Annual rates of decline in pregnancy-related mortality for the ≤19 years and 20-29 years age-groups were 4% and 2% respectively over the 1976–2005 period, both of which were statistically significant (p<0.05) (Fig. 3).

Also, there has been a reduction in maternal deaths among those with birth-order 1-3 (Fig. 3). For the first-order pregnancy, the mortality rate of 924 per 100,000 pregnancies in 1976–1985 declined to 280 per 100,000 pregnancies in 1996–2005. For the second- and third-order pregnancies, the corresponding reduction was from 328 to 186 per 100,000 pregnancies. Annual rates of decline in pregnancy-related mortality for the first-, and the second- and third-order pregnancies (combined) were 6% and 3% respectively, both of which were statistically significant (p<0.05). However, for higher than the third-order pregnancy, there was no apparent trend towards a change in mortality during 1976–2005.

In 1996–2005, the likelihood of a maternal death in the first-order pregnancy declined by about 70% compared to that in 1976–1985. The corresponding decline for the second- and third-order pregnancies was 43%. As the total fertility rate declined over the time period, the percentage of first birth has increased; however, the percentage of first births below 19 years of age decreased over this same period in both ICDDR, B and government service areas. We can assume that the higher ages at first births may have impacted at the national level as well. There also has been a shift from high-parity birth to low-parity birth. However, based on Matlab data, we did not find any significant effect of this shift on the decline in the MMR.

### Indicator 2—Increase the proportion of births attended by skilled health personnel

#### Rates of use of professional birth attendants

**Fig. 4:**
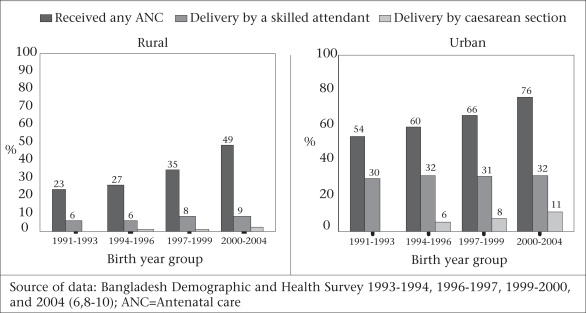
Trends in the use of maternal health services, Bangladesh, 1991–2004

Despite improvements in maternal mortality, Bangladesh remains a country of massive deprivation: during 2000–2004 when the MMR was 322, only 13% of delivering-women used professional care for birthing, and 9% of births were in facilities (6). By 2007, these rates had improved: 18% were reported delivering with professional care and 15% were in facilities (7). Use-rates of skilled birth attendants in rural areas have increased significantly from 5.6% during 1991–1993 and 9.0% during 2000–2003 to 13% in 2005–2007. Rates in urban areas have only recently improved, reaching 36.5% in 2005–2007, from a plateau at about 30% recorded since 1991–1993 (Fig. 4) (6-10). Given these relatively low use-rates of professional care providers in both rural and urban areas, the traditional birth attendant remains the primary birth care provider. The increase in professional care in rural areas since 1993 has been witnessed in the public, private and NGO-sector facilities; there has been no significant increase in delivery by home-based skilled attendants.

#### Caesarean section rates

**Fig. 5:**
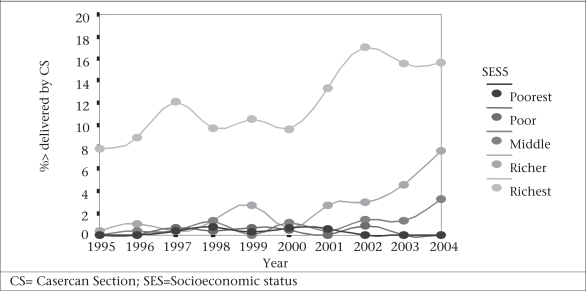
Inequalities in delivery by caeserean sections Bangladesh, 1995–2004

Rates of the use of caesarean section are increasing primarily for the least poor, well-educated urban woman (Fig. 4). In rural areas, the rate increased from 0.9% to 1.7% from 1995–1996 to 2000–2004 and then to 5.4% in 2005–2007 while in urban areas, the corresponding rates doubled—from 5.6% to 11.4% and then increased to 16.2% in 2005–2007. Only the two least poor quintiles achieved greater than a 3% use-rate—with 14% use-rate of caesarean section in 2004 being the highest level of use (Fig. 5).

Obstetric surgery is a necessary component of the safe motherhood services, but it can also lead to exploitation of women and families due to the emergency nature of some complications requiring expensive surgery. Nearly half of the deliveries in private facilities during 2001–2003 were caesarean (1). In a 12-district study in 2005, this proportion was even higher (70%) (Anwar I. Personal communication, 2006). This trend bears watching, as medi-cal need is not the primary reason for high rates of private ceasarean-section use in other countries, such as Egypt and Brazil (11,12). Possible financial exploitation is only one problem; another is that reversal of the caesarean section trend has only marginal success in other countries (13-15).

**Fig. 6:**
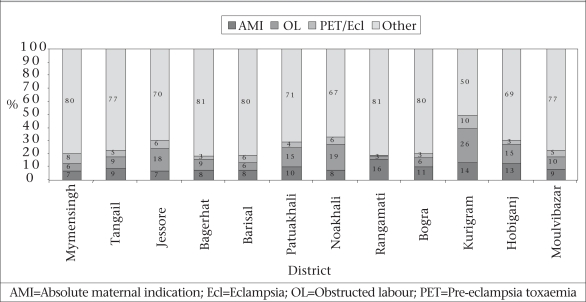
Obstetric surgeries by indication per district

The indicator—‘met need for obstetric care’—attempts to measure the extent to which caesarean sections and other obstetric surgeries are performed for life-threatening situations. In the 12-district study of 2005, only 7-16% of all obstetric surgeries carried out in public, private and non-governmental facilities were due to life-threatening obstetric conditions (Fig. 6). Life-threatening conditions include placenta previa, placenta abruptio, transverse/oblique lie, face/brow presentation, major mechanical cephalo-pelvic disproportion, and ruptured uterus. Even if surgeries for all obstructed labour and eclampsia/severe pre-eclampsia patients are added to this list, 50-81% of obstetric surgeries were performed for other maternal and foetal indications (Botlero. Personal communication, 2006). Most common among these indications are ‘post-dates’ and ‘previous caesarean section’.

This same indicator—‘met need for obstetric surgeries’—quantifies the gap in access to life-saving surgery by residence. Women residing outside city centres in the study subdistricts suffered a huge shortfall in live-saving obstetric interventions. Most rural areas of the districts fell below the estimated 0.7% of pregnant women who would die without recourse to life-saving surgery (2; Botlero. Personal communication, 2006).

Thus, increases in caesarean-section rates are observed in private clinics with relatively wealthier women who may not require it for medical indications. Given this, the measure of caesarean-section rates looses its value both as a measure of improvements in maternity services and as a measure of equity. The alternative measure of the ‘met need for obstetric care’ is a much better indicator of improvements in maternity care and for monitoring regional and equity needs.

#### Use of antenatal care

**Fig. 7:**
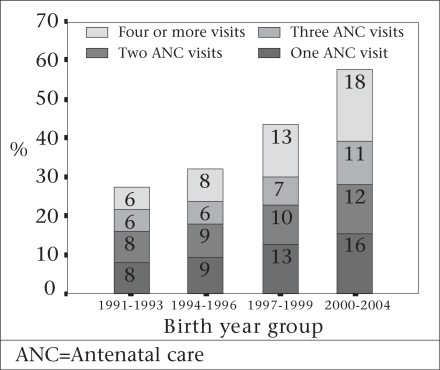
Trends in number of antenatal care visits, Bangladesh, 1991–2004

The increase in the use of antenatal care has shown promise—the proportion of women who had at least one antenatal care consultation more than doubled over the 17 years for which there are data—from 27% in 1991–1994 to 60% in 2005–2007 (6-10). The increase was greater for the fourth antenatal care visit than the first, the second, or the third visit (Fig. 7). Area of residence, socioeconomic status, higher education of mother, and lower parity are significant predictors for seeking antenatal care (1,6,7).

#### Use of postpartum care

Much less is known about the use of postpartum care, the importance of which has only recently become a concern given that most maternal and newborn deaths occur within 48 hours of delivery. The use of a skilled attendant at birth should address the vulnerability at this time period. However, the use of postpartum care with a skilled care provider in Bangladesh remains limited with 21% using such care during 2005–2007, most (20%) within the first 48 hours after birth (7).

#### Inequities over time

**Fig. 8:**
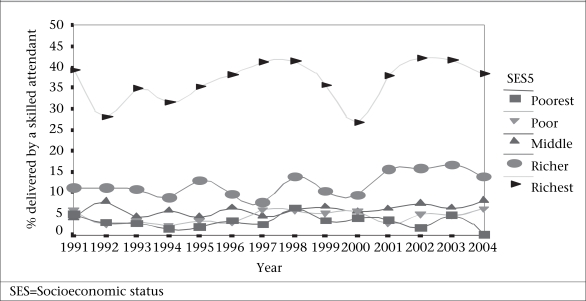
Trends in inequalities in accessing skilled attendance in Bangladesh

**Fig. 9:**
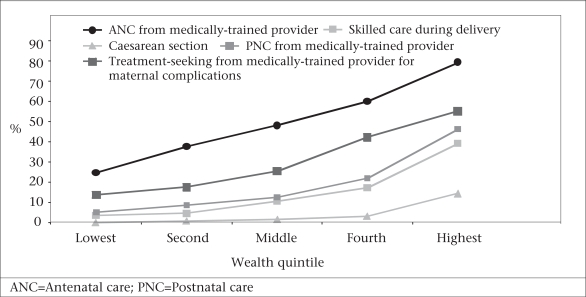
Bangladesh: use of maternal health service indicators by the wealth quintile, 2001–2004

Over the 14 years for which there are data, 60% of the population that makes up the lowest three asset quintiles were non-recipients of services—whether services mean skilled birth attendants, delivery at facilities, or by caesarean section (Fig. 8). The movement towards professional care at birth in Bangladesh has kept pace with the increase in births but has shown no progress beyond that. Many gaps in coverage obviously exist throughout the country. Those excluded from services continue to be at risk, and the inequalities between the rich and poor, the urban and rural, continue to grow. The major challenge remains in rural areas, although urban areas are also in need. These are areas of ‘massive deprivation’ where only the rich escape exclusion from care (Fig. 9).

#### Strategies to increase uptake of services

Bangladesh faces an array of barriers to the uptake of professional birthing care. Reluctance to use services and poor-quality care detract from use where services are available while the lack of care providers, supportive infrastructure, and policies are the primary barriers for women in the sites of massive deprivation. This section addresses these access issues where services are available but underused and reviews the constraints to scaling up where there are currently not enough services.

#### Predictors of use and perceived barriers

Some predictors of care-seeking are obvious—those who live in urban areas, who are least poor, are educated, and who use services more—whether it be antenatal care, skilled birthing care, facilities for delivery, or caesarean sections. From a multivariate analysis of survey data collected during 1991–2004, other significant predictors for the use of skilled attendants or caesarean section include the number of antenatal care visits, higher education (not only of mother but also of father), division of residence (Khulna being the outstanding division), lower parity, and singleton birth (6,8-10).

When asked, 91% of women stated that they did not use a facility for birth because they did not feel any need for it (68%), costs are involved (18%), and it is ‘not customary’ (9%). These reasons are the same as given by 83% of women for not seeking postpartum care, and the first two are the same for those not seeking antenatal care (1). Surprisingly, fears of services or not wanting to be attended by a male physician during birth were mentioned by less than 5% of women.

In Matlab where the use of facilities for delivery was over twice the national level at the time of the study (about 21% in 2001), women mentioned other barriers: fear of referrals to district-level facilities, with their attendant costs being their number one barrier. Other barriers, starting with the most mentioned, are influence of husband, indirect costs, concerns that delivery may take place on the way, distance to facility, household responsibilities, and previous bad experiences with the service providers due to their behaviours (16).

### Addressing barriers

### Knowledge of danger signs/birth-preparedness

The primary barriers to the uptake of care as perceived by women throughout Bangladesh are clear: no feeling of need for skilled care at delivery and high costs that may face her and her family when a complication is recognized. The demand for skilled care corresponds primarily to the perception of complications. Yet, recognition of danger signs for which a woman should seek care is a barrier the world over: most obstetric complications that kill are a continuum of normal birthing—prolonged labour only becomes a problem after the woman has been in normal labour for 12 hours; postpartum bleeding is a problem after it becomes excessive (>500 mL). Bangladesh—both urban and rural—is no exception: awareness of danger signs is low but better for prolonged/obstructed labour (about 50% in both rural and urban areas) and tetanus (nearly 60% in both the areas) than for the primary killers—eclampsia (about 30%) and excessive bleeding (26% in urban areas and 20% in rural areas) (6). Even with low awareness, about a quarter of women stated that they had suffered at least one of the five major obstetric complications in a recent pregnancy: 17% with prolonged labour of over 12 hours and 11% with excessive bleeding. Of those who perceived they had a life-threatening complication, only a third sought care from a qualified provider and another third from unqualified providers (6). Costs are the primary reason keeping the woman away from care (44%), although over a third also mentioned that the condition was not serious or treatment was not necessary.

Qualitative efforts bear out this care-seeking pattern. In a semi-qualitative survey of nearly 1,500 women in Sylhet (17), half of them reported a ‘serious’ complication during their last pregnancy and/or delivery with 86% seeking care. However, the care sought was to bring a treatment to the home in the majority (68%) of cases. This care consisted of tablets, spiritual water and/or oil, homeopathic medicines, and allopathic medicines provided by untrained pharmacists or medically-trained local doctors. When they did seek care outside the home (30%), 11% went directly to EmOC facilities, and another 11% went to a health clinic or the home of a medically-trained provider. The message from this study is that care-seeking for complications, a major part of any safe motherhood strategy, can have local meanings that thwart the intended effort to guide women to skilled care rapidly. And the need for skilled care for normal births because all births are at risk of a complication, is not even considered by women.

Addressing these household/community/cultural barriers has only just begun in the Bangladesh public-sector programme at the community level. The GoB's skilled birth attendants at the community level (CSBAs) can be a primary source of information as they reach into households with messages on birth-preparedness (recognizing danger signs, where to go, and financing possibilities). However, with only 2,500 CSBAs for a country with 3.25 million births in rural areas per year, this strategy is still very limited.

Antenatal care is an obvious time to receive birth-preparedness information. With 60% of women receiving antenatal care, primarily with qualified doctors (35.5%) and nurse/midwife/paramedics (16%), there is real potential to build on this strategy (7). That potential still needs to be realized: as of 2004, only three in 10 women with a livebirth in the five years preceding the survey received information on signs of pregnancy-related complications during antenatal care from any provider (6). As the use of antenatal care increases, it obviously presents the primary means by which to talk with women about their intentions regarding birth and to provide birth-preparedness information. Special efforts will be needed to ensure that the poorest women use antenatal care and receive the messages.

### Costs of care

A major constraint to the use of maternal healthcare is the cost of delivery, the fear of costs (especially for a complicated delivery), and the inability to find money when needed. This latter constraint is particularly difficult for those working seasonally (e.g. agriculture), but resource constraints are not limited to rural areas. About half of the families in urban Bangladesh did not have enough cash for a normal delivery, and three-quarters did not have enough for a caesarean section in urban Sylhet. Delays caused by searching for money can have serious implications for maternal and newborn outcomes (Moran A. Personal communication, 2006).

For those who use services, the costs involved in birth even in the public sector are high. Charging starts at the hospital gate and is followed by charges for medications, diagnostic tests, and ‘tips’ to ‘manage’ at all levels (18). These unofficial costs are exacerbated for delivery—the single most costly event across the pregnancy period, followed by postpartum care; complicated deliveries are even more expensive involving costs to households between three and 10 times more than normal deliveries (19). The cost of complicated deliveries is often ‘catastrophic’, defined as exceeding 10% of annual household income (20). However, there appears to be no significant difference in the amount paid for a normal delivery in hospital by the wealthy group (19).

To address these problems, different NGOs and the ICDDR, B have initiated many financing schemes, primarily through community insurance, on a small scale in Bangladesh. The public sector has recently initiated a voucher scheme with donor support to offset the costs of delivery (in public or private facilities) for the poorest. Vouchers target specific marginalized groups who can redeem the vouchers in exchange of free maternal services in health facilities contracted in advance by the voucher agency. They overcome the problem of access to cash by avoiding upfront payment for care, limiting household expenditure to transport costs (and time). Since these innovative financing schemes have only begun, evaluations are not yet available from Bangladesh; so, their impact on maternal healthcare cannot be quantified.

## Barriers to quality of care

For women who use services, the quality of care is a major challenge. At the very least, provision of care should not inhibit use and be at a standard that results in the best possible outcome given available resources. However, results of studies in countries as varied as Rwanda and Jamaica, Ecuador and Benin, showed that credentials, whether of a doctor, a nurse, or a midwife, did not guarantee a skilled provider. Tested or observed, scores for skills among providers with credentials have been surprisingly low for normal care, safe delivery, and newborn care (21-27) and do not measure up to ‘trained-to-proficiency’ where an agreed level has been set (28). There have been no observational studies of providers with credentials in Bangladesh; however, it is unlikely they would fare differently. Even if more women access care with health professionals for their pregnancy, a few are likely to receive clinical care of an adequate standard.

Although professional providers with longer training fare poorly on evaluations, the more controversial community workers, a stopgap measure selected by many countries with massive deprivation, appear to do better on evaluation. The Bangladesh's CSBAs with 21 weeks training and Nepal's maternal and child health workers (MCHWs) with six months training are examples. Testing in both Nepal (29) and Bangladesh (30) found relatively high levels of knowledge, skills, and performance for these workers. The differences in tested skill levels between credentialed and community providers may be due to variations in tests, interim since trained, training time, training objectives, or curricula.

The other quality-of-care indicators at facility level are the use of active management of the third stage of labour (AMTSL) and the use of magnesium sulphate (for eclampsia)—two evidence-based practices. In four districts of Bangladesh, it was found that, even in districts with higher use-rates, only 64% and 70% of personnel in the public, private and NGO facilities stated that they used AMTSL and magnesium sulphate in the last three months respectively. The use of AMTSL is particularly deficient—only 50% of the district, subdistrict and medical college hospitals used AMTSL, although the evidence of its effectiveness has been available for more than 20 years.

Referral—another important aspect of quality maternal care—should facilitate access to the most appropriate level of care and improve the efficiency of services facing resource constraints. At the subdistrict level, referral is typically made to hospitals at the district level and at the medical colleges, but patients often opt for private clinics and hospitals anticipating better quality services. On inspection of technical quality (e.g. infection-prevention practices, available instruments and equipment, discharge advice, laboratory and blood supply, signal functions) in private facilities in four districts, the quality was found to be poorer than that provided at NGO and public facilities (Anwar I. Personal communication, 2006).

In Bangladesh with its low coverage for professional delivery care, the strategy to improve the quality of care is to provide refresher training for some credentialed providers and longer training (one year) for skills in surgery and anaesthesia. However, much more is needed. Systems need to be strengthened in terms of management and logistics, personnel in terms of technical and caring capacity, and levels of care connected and made accountable as a total system providing the continuum of care.

### Critical barrier—access

Access to skilled care is not considered a major problem by women: only 12% of those who thought that they had a life-threatening problem and 6% of those with no such problem stated that access was a problem (1). Access problems include ‘too far’, ‘no one to accompany’, ‘do not know where to go’, and ‘do not know how to go’ (1), although women's perception that no care is needed for maternity may outweigh other considerations. Yet, access to skilled birthing care is a major problem in Bangladesh.

Throughout rural Bangladesh, skilled staff to manage normal and complicated deliveries is at a premium. There are three ways to look at the deficit:

- In the public sector, as of the end of 2005, there were at best approximately 1,500 CSBAs trained plus 5,000 Family Welfare Visitors (trained prior to 1994) to manage the estimated 3.25 million births in rural areas per year—only one SBA for every 500 births per year. To manage the approximate half a million complicated births are 128 obstetricians, 142 medical officers trained in obstetrics for one year available in the public sector (31), and about 60 trained doctors in the Maternal and Child Welfare Centres (MCWs)—or one per 1,500 cases per year.
- Doctors managed nearly 7.5% of births according to the health and demographic survey 2004 (6). If this doctor had training in obstetrics, it would mean that one trained doctor attended nearly 900 births in that year. Students of medicine now have the option of selecting surgery or obstetrics during their training; most select surgery and, hence, have little experience in obstetrics. Clearly, doctors without any training in obstetrics are attending deliveries. Only 5.7% of births were managed by nurses/midwives/FWVs which implies one nurse/midwife attending about 30 births per year (6). While this is doable, an increasing coverage may be difficult.
- A third way of looking at the coverage and related issues is to analyze hospitals for their ability to function as emergency obstetric care facilities. In the GoB programmes (HPSP and HNPSP), all 13 medical college hospitals, 59 district hospitals, 64 of the 90 MCWCs, and 120 of the 405 rural subdistrict hospitals were to be upgraded with renovations, logistics/equipment, and personnel to provide emergency obstetric care. By mid-2005, however, only 70 (17% of all) subdistrict hospitals and 25% of district hospitals had a team of medical officers trained in obstetrics and anaesthesiology in upgraded facilities (31).

Evidence from many developing countries suggests that the sheer lack of staff and facilities is the most significant barrier to progress towards the increased use of skilled birth care. Where professional skilled care is not available, coverage gaps cannot be remedied without tackling major health system issues—increasing the supply of providers through training, providing incentives for trained providers to live and provide services in places of need, upgrading facilities and supply systems to ensure safe births, and ensuring the management capacity, policies, and regulations to support the providers.

### Increasing the supply of skilled care providers

The supply of professional skilled birthing care depends on strategic decisions in three areas: training, deployment, and retention of health workers, and these factors, taken together, determine the coverage of birthing care.

Birthing care requires skills and clinical judgment that come only with knowledge and practice. Questions that need to be addressed by policy-makers include: whom to train, duration of training, and the numbers of births to be managed during that time for students to reach proficiency in required skills. Based on a survey of members in 22 countries, the International Confederation of Midwives states that theory and clinical learning to become a skilled birth provider each requires approximately one year (32). However, where the use of skilled birth attendants is low, rapid start up to increase coverage is hampered by limited facility-based deliveries for trainees and lack of training instructors and training institutions. Each midwifery student should manage 40-50 births during training with supervision, yet poor countries, such as Bangladesh, typically lack the number of facility-based births needed. Standards may be lowered to accommodate larger numbers of students.

Posting choices include location where providers deliver services (homes, health centres, or hospitals with beds for normal deliveries, hospitals for referral deliveries), responsibilities (multi-purpose versus dedicated; basic tasks or life-saving tasks as well), and provision through teamwork or solo.

In facilities, the norm is teams of providers to attend births, most often composed of a mix of professionals and non-professionals. The advantages of birthing teams in facilities are obvious: skilled providers can provide care for (or oversee) a larger number of patients simultaneously, and working hours are more regular. Midwives in Matlab stated that they highly prefer facility-based birthing over home birthing as they are in charge: they can ensure safety, cleanliness, and availability of supplies, accommodate other work, facilitate referrals easier, and call on clinical colleagues and emergency transport if needed (16).

In home-births, a single care provider typically attends. In homes, families take charge—pushing cultural norms, such as injections to hasten delivery, or refusing referrals and questioning the credibility of the midwife for such a suggestion. The midwives in Matlab felt a lack of control over their time, space, and safety, especially at night (16).

Regulations concerning what midwives, nurses, and community health workers can provide to save lives are important, especially where solo workers and long distances from referral sites are the norm. Delegation of responsibility to professional providers to give safe quality care beyond the traditional scope of their specialty has succeeded through policy plus competency-based training. In Bangladesh, regulations have extended the scope of work of many first-line providers. For example, CSBAs are allowed to provide the first dose of magnesium sulphate to stop convulsions prior to referral. Medical officers trained in surgery for one-year can conduct caesarean sections. However, the need for anaesthetists has not yet found such a remedy, and they remain in short supply.

Retention of providers is a major part of the supply problem. While the marked migration and death of providers from HIV/AIDS known in many sub-Saharan countries are not problems in Bangladesh, but maldistribution of providers is. As in other South and Southeast Asian countries, rural postings go unfilled for reasons of inadequate income, low prestige, poor rural infrastructure for children, and social isolation. Government attempts to address maldistribution, with bonds on clinical graduates to work in rural areas, have achieved only minor success as transfers are easily purchased, resulting in the rural shortfall in professional care. Good management practices and the highest level of commitment by politicians and professionals are much needed.

### Options for increasing coverage where supply is constrained

Given that improved training, deployment, and retention are crucial tools with which to break the supply barrier, we model six possible scenarios based on Bangladesh data for population, births per year, and training capacity to determine the possible coverage in 2015. Variables include training time for CSBAs (6 vs 18 months) and posting site (home vs facility) anticipating that those who work in the home work solo while those in facilities work in teams. Attrition is set at 10% per year—a conservative rate given the attrition reported in the public sector in Bangladesh. The options are as follows:

- Train female community-based health assistants and family welfare assistants in midwifery (midwife assistants) for six months to work solo with deliveries at home. They should link women with back-up care. Based on district data, such workers cover about 20 deliveries per year.
- The same as above but anticipate coverage at 50 deliveries per year.
- Train community workers (called ‘midwives’) for 18 months to provide care in facility-based teams with other midwives. Based on data from 87 hospitals in African NGOs, each midwife in such a facility could cover approximately 175 births per year (33).
- Similar to ‘c’ but each midwife covers about 220 births per year based on observational data from African hospitals (34).
- Mixed teams to include one midwife for every two midwife assistants who together cover 175 births per year per midwife and per midwife assistant.
- Mixed teams to include one midwife for every two midwife assistants who together cover 220 births per year per midwife and per midwife assistant.

The team size in model ‘e’ and model ‘f’ is not known. To a large extent, the team size will depend on the geographic dispersion of the population and the infrastructure and resources available. It is anticipated that a team of 3-4 midwives with double the number of midwife assistants in a facility is the minimum to allow for the coverage of 24 hours a day, seven days a week.

To improve the use of skilled birthing care, these options need to be complemented by initiatives that aim to strengthen individual, family, and community behaviours—both in terms of self-care and improved care-seeking.

Taken over a 10-year period from 2005 to 2015, Figure 10 shows that the gain in coverage is highly dependent on the numbers of births carried out per provider per year. The key to increasing the numbers of births per provider is a deployment choice: working in a facility in a team allows midwives to cover far more births than working solo for deliveries at home. A mixed team with midwives and midwife assistants achieves a higher coverage faster because more teams with this composition can be produced than teams of only midwives with 18 months’ or more training.

**Fig. 10:**
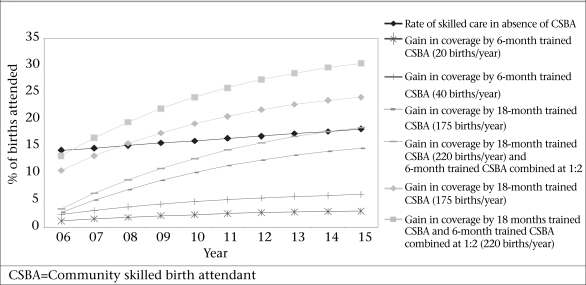
Skilled care at delivery: projected scenario, 2006–2015

Facility-based birthing with mixed teams increases the coverage over a 10-year period by 24-30% of births. This means theoretically that, by 2015, facility teams, carrying out 220 births per year per team member, could achieve nearly 50% coverage given that the present trajectory without any improvements would achieve nearly 20% coverage in 2015. This compares well with a midwife assistant covering deliveries in the home who carries out only 20 births per year (gain of 3% coverage) or 40 births per year (gain of 6%). Even if the training output was doubled (the Bangladesh training capacity now allows 1,000 trainees per year), these percentages would only double—meaning the facility-based teams still would cover far more births than individual providers attending births at home. Levels of retention highly influence the levels of coverage that can be reached. If the loss per year were 5% instead of 10%, the coverage rates could be raised by over 25%.

Providing facility-based birthing care can be more cost-efficient than births at home. Costs to the health system for provision of delivery care at home or at a health centre depend on the case-load: where case-loads at facility are higher, facili-ty-based care becomes less expensive than even deliveries at home (in rural Bangladesh, the breakpoint was 136 births per year in the health centre) (19). Costs to the Bangladeshi families are about the same for a birth with a midwife at home or at a health centre—less than 10% of the husband's monthly salary. In contrast, normal deliveries at a hospital cost 2-3 months’ income of the husband (public vs private facility respectively) (35).

## CONCLUSIONS

Bangladesh is on its way to achieving MDG 5 in terms of reducing maternal mortality, but the rate of decline needs to speed up over the next decade. The initial downward trend is the likely product of the decline in fertility, shifts in the risk groups towards those less likely to die (younger but not too young), and reduction in abortion-related deaths. Further reductions are less likely to come with further decreases in fertility unless the age of first birth can be shifted to a less risky age-group (20-29 years). Little is known about the national level of abortion-related deaths—whether improvements in the quantity and quality of menstrual regulation services could improve maternal outcomes.

However, it is assumed that improvements in the use of skilled birthing care and appropriate and timely EmOC use would make a difference. The low level of increase in such use over the last 17 years, except for the use of caesarean section, needs to be addressed, not only with increases in skilled care available close to women, but with convincing messages to women and the decision-makers in their families, concerning the need for using such services. Antenatal care use is relatively high, even among the most poor and is the most likely avenue by which to transmit such messages. Beyond messages, financing for delivery care counts, and families obviously anticipate that deliveries at the facility will cost them money they may not have. Ensuring that services are available, especially for the poorest is paramount—either through vouchers or community financing. More such means need to be tested.

The GoB has been trying to improve maternal care over the last decade; however, these strategies have yet to realize their potential. More than a decade later, the EmOC strategy has resulted in only 17% of health facilities at the subdistrict level being adequately staffed (about 50% of those targeted). At the district level, only 25% of the district hospitals are ready, although most MCWCS (also at the district level) provide comprehensive obstetric services. The retention of skilled birth attendants, specifically obstetricians and anaesthetists, at the rural upazila hospital level, is key and demands more attention at policy and programme levels.

The CSBA strategy, whether trained for six or 18 months, is unlikely to increase coverage to the levels targeted within the stated timeframe (50% by 2010). The coverage is more likely to increase, if the CSBAs do team work in health centres so that they can cover over 150 births per year each.

## RECOMMENDATIONS

Expanding the coverage rapidly requires training of midwives and deployment preferably in teams and small facilities. Based on simulations, teams working in facilities will increase the coverage by up to 30% by 2015. Having providers in teams is the efficient option, it provides the possibility of scaling up as much as 10 times more quickly than deploying solo health workers in deliveries at home.

The most likely and obvious scenario is to bring CSBAs together with retrained Family Welfare Visitors (FWVs) at union-level clinics. Even with one FWV and two CSBAs, there may be increases in coverage for delivery care. Stimulation of women and their families during antenatal care at satellite clinics to use such skilled delivery care needs to be tested.

To ensure that facility-based EmOC is available and responsibly provided, mechanisms for accountability need to be put in place. Yet, the only group without a conflict of interest may be that at the community level—consumer groups as watch dogs. There is no precedent for such consumer groups in Bangladesh, although they have been successfully formed in other countries. Professional associations could also provide such oversight—and have done so in terms of reviewing statistics per facility to date. However, actions to improve the quality of care have not been taken.

The way forward to achieve MDG 5 in Bangladesh depends on improvements in the health systems. This is not just a technical debate; it will require the buy-in from top political stakeholders to improve facilities and staff and supply them adequately. Moving to this level of debate is the next step in achieving MDG 5.

## Assumptions and basis of calculations

Population growth rate: 0.019/person/year (1.9/100 population/year)

Formula for calculating projected population: P_t_=P_0_e^rt^

Census date: 22 January 2001. Population projected for the date of 30 June of the relevant years to ease calculation on the basis of mid-year population); population on 22 January 2001: 123,851,000

Crude birth rate: 27/1,000 population [UNICEF]; stillbirth rate: 37/1,000 pregnancies [BDHS 2004]; Assumption: 37 stillbirths for 963 livebirths [twin/triplets pregnancies are not taken into account]

Rate of delivery at the facility has been calculated on the basis of linear regression of the data from 1993, 1996, 1999, 2000, and 2003 DHS of Bangladesh [constant:3.009; regression coefficient: 0.648]

Attrition factor for the stock of SBA: 10%

The Government is planning for an 18-month training of SBAs [6 months training at first (4 months at facility + 2 months at the community) + 9 months on-the-job placement in the community + 3 months’ refresher training based on the performance during on-the-job placement). In that case, our assumption is that we will be able to produce 600 SBAs/year, instead of 1,000/year using all facilities in 40 districts

The number of deliveries has been considered as equal to the number of births (live + still) ignoring twins and triplets

Numbers have been rounded in the case of decimal places

## Footnotes

*The Matlab field area is divided into two areas, known as the MCH-FP intervention and government areas. ICDDR, B provides health services in the MCH-FP area, and the Ministry of Health and Family Welfare provides services in the government areas; FP=Family planning; MCH=Maternal and child health

## References

[1] (2003). National Institute of Population Research and Training. Bangladesh maternal health service and mortality survey.

[2] Dieltiens G, Dewan H, Botlero R, Biswas K, Begum SN, Shahjahan M (2005). Met need for life-saving obstetric surgery in Bangladesh. the Matlab-ICDDR, B cohort study of maternal mortality 1990–2001 & the results for a new indicator to assess met need for life-saving obstetric surgery.

[3] Fauveau V, Blanchet T (1989). Deaths from injuries and induced abortion among rural Bangladeshi women. Soc Sci Med.

[4] Ronsmans C, Khlat M (1999). Adolescence and risk of violent death during pregnancy in Matlab, Bangladesh. Lancet.

[5] Fauveau V, Koenig M, Wojtyniak B, Chakraborty J (1988). Impact of a family planning and health services programme on adult female mortality. Health Pol Plann.

[6] (2004). National Institute of Population Research and Training. Bangladesh demographic and health survey 2004: preliminary report.

[7] (2007). National Institute of Population Research and Training. Bangladesh demographic and health survey 2007: preliminary report.

[8] Mitra S, Ali M, Islam S, Cross A, Saha T (1994). Bangladesh demographic and health survey 1993–1994. preliminary report.

[9] Mitra S, Al-Sabir A, Cross A, Jamil K (1997). Bangladesh demographic and health survey, 1996–1997.

[10] (2001). National Institute of Population Research and Training. Bangladesh demographic health survey 1999–2000.

[11] Khawaja M, Kabakian-Khasholian T, Jurdi R (2004). Determinants of caesarean section in Egypt: evidence from the demographic and health survey. Health Policy.

[12] Potter J, Berquo E, Perpetuo I, Leal O, Hopkins K, Souza M (2001). Unwanted caesarean sections among public and private patients in Brazil: prospective study. Br Med J.

[13] Notzon F, Cnattingius S, Bergsjo P, Cole S, Taffel S, Irgens L (1994). Cesarean section delivery in the 1980s: international comparison by indication. Am J Obstet Gynecol.

[14] Sloan N, Pinto E, Calle A, Langer A, Winikoff B, Fassihian G (2000). Reduction of the cesarean delivery rate in Ecuador. Int J Gynaecol Obstet.

[15] Flamm B, Berwick D, Kabcenell A (1998). Reducing cesarean section rates safely: lessons from a “breakthrough series” collaborative. Birth.

[16] Blum L, Sharmin T, Ronsmans C (2006). Providing home-based and facility-based basic obstetric care: understanding the perspective of skilled birth attendants. Reprod Health Matters.

[17] Moran A, Winch P, Sultana N, Kalim N, Afzal K, Koblinsky M (2007). Bangladesh PROJAHNMO Maternal Morbidity Study Group (1&2). Patterns of maternal care seeking behaviors in rural Bangladesh. Trop Med Int Health.

[18] Afsana K (2004). The tremendous cost of seeking hospital obstetric care in Bangladesh. Reprod Health Matters.

[19] Borghi J, Ensor T, Somanathan A, Lissner C, Mills A (2006). Lancet Maternal survival Series steering group. Mobilising financial resources for maternal health. Lancet.

[20] Ranson K (2002). Reduction of catastrophic health care expenditures by a community-based health insurance scheme in Gujrat, India: current experiences and challenges. Bull World Health Organ.

[21] Adeyi O, Morrow R (1997). Essential obstetric care: assessment and determinants of quality. Soc Sci Med.

[22] Harvey S, Ayabaca P, Bucagu M, Djibrina S, Edson W, Gbangbade S (2004). Skilled birth attendant competence: an initial assessment in four countries, and implications for the Safe Motherhood movement. Int J Gynaecol Obstet.

[23] Hermida J, Robalino ME (2002). Increasing compliance with maternal and child care quality standards in Ecuador. Int J Quality Health Care.

[24] Miller S, Cordero M, Coleman A, Figueroa J, Brito-Anderson S, Dabagh R (2003). Quality of care in institutionalized deliveries: the paradox of the Dominican Republic. Int J Gynaecol Obstet.

[25] Ruano A, Gonzales R, Gilson G (1999). Training evaluation report. Mother Care/Guatemala.

[26] Ugalde M, Conover C, McDermott J (1999). Training evaluation report. MotherCare/Bolivia.

[27] Marquez L Baseline assessments of essential obstetric care: Bolivia, Ecuador, and Honduras. Bethesda, MD: Quality Assurance Project, 2001. (www.qaproject.org, accessed on 3 June 2006).

[28] McDermott J (1999). Training evaluation report. MotherCare/Indonesia.

[29] Carlough M, McCall M (2005). Skilled birth attendance: what does it mean and how can it be measured? A clinical skills assessment of maternal and child health workers in Nepal. Int J Gynaecol Obstet.

[30] (2004). World Health Organization. Skilled birth attendance: review of evidence in Bangladesh.

[31] (2005). Management Information System—Health. Human resources and EmOC performances (Voice of MIS-Health. Newsletter 1).

[32] Fullerton J, Severino R, Brogan K, Thompson J (2003). The International Confederation of Midwives' study of essential competencies of midwifery practice. Midwifery.

[33] Van Lerberghe W, Van Balen H (1992). Typoligie et performances des hopitaux de premier recourse en Afrique sub-Saharienne.

[34] Jaffre Y, De Sardan O (2004). Une medecine inhospitaliere: les difficiles relations entre soignants et soignes dans cinq capitals d'Afrique de l'Ouest.

[35] Borghi J, Sabina N, Blum LS, Hoque ME, Ronsmans C (2006). Household costs of healthcare during pregnancy, delivery, and the postpartum period: a case study from Matlab, Bangladesh. J Health Popul Nutr.

